# The contemporaneous epidemic of chronic, copper deficiency

**DOI:** 10.1017/jns.2022.83

**Published:** 2022-10-11

**Authors:** Leslie M. Klevay

**Affiliations:** Internal Medicine, University of North Dakota, School of Medicine and Health Sciences, Grand Forks, ND 58202-9037, USA

**Keywords:** Alzheimer's disease, Copper deficiency, Ischaemic heart disease, Myelodysplastic syndrome, Osteoporosis, Peripheral neuropathy

## Abstract

The classical deficiency diseases have nearly disappeared from the industrialised world and are thought to be found largely in sub-Saharan Africa and South Asia. More than 80 collected medical articles, mostly from Europe and North America, describe more than 9000 people with low concentrations of copper in organs or tissues or impaired metabolic pathways dependent on copper. More than a dozen articles reveal improved anatomy, chemistry or physiology in more than 1000 patients from supplements containing copper. These criteria are diagnostic of deficiency according to The Oxford Textbook of Medicine. Alzheimer's disease, ischaemic heart disease and osteoporosis receive major emphasis here. However, impaired vision, myelodysplastic syndrome and peripheral neuropathy are mentioned. Copper deficiency probably causes some common, contemporaneous diseases. Advice is provided about opportunities for research. Seemingly authoritative statements concerning the rarity of nutritional deficiency in developed countries are wrong.

## Introduction

The classical deficiency diseases (beriberi, pellagra and scurvy) have disappeared, almost. For example, in 1974, there were three deaths from scurvy in the United States^(1)^. Prominent publications in the field of nutrition express the opinion that nutritional deficiency is rare in developed countries.

Deficiency diseases are rare in the general population^(2)^. ‘Nutritional deficiency disease is clearly not a major public health problem’^(3)^. Deficiency diseases are thought to be found largely in sub-Saharan Africa and South Asia^(4)^. To my knowledge, there are no recent, large nutrition surveys similar to those we did in Latin America and the United States a half century ago; copper was not evaluated. It was suggested that (nutritional) standards for preventing deficiencies are different from those for preventing chronic disease^(5)^.

Osler^(6)^ described ‘severe and chronic forms’ of pellagra more than a century ago. Thus, deficiency can be chronic; it can develop slowly^(7)^, especially with small nutrient deficits^(8)^.

## Defining and discovering nutritional deficiency

Dann and Darby^(9)^ defined five zones of nutriture shown in Table 1. Their concepts were extended to trace elements with examples related to copper and zinc in animals and people^(10)^. According to Golden in the Oxford Textbook of Medicine^(11)^, low nutrient intakes can reduce nutrient concentrations in tissues and compromise metabolic pathways. Diagnosis is relatively straightforward with measurement of tissue nutrient or by testing metabolic pathways. A beneficial effect on a metabolic pathway or functional system from nutrient replacement is a sign of deficiency; this effect is the characteristic of zone four^(10)^.

**Table 1.** Defining and measuring nutritional status^(9^^)^

1.	Saturation
2.	Unsaturated but functionally unimpaired
3.	Potential deficiency disease
4.	Latent deficiency disease
5.	Clinically manifested deficiency disease

It will be affirmed here that evidence from numerous medical articles supports the belief that copper deficiency contributes to some common, Western diseases according to Burkitt's terminology^(12)^. These diseases are seemingly new, having arisen largely in the 20th century, and are associated with affluence, industrialisation and habitual consumption of diets high in animal protein, fat and refined sugars and low in fibre, phytic acid and starch^(13)^. This diet often is low in copper^(13)^.

Some of these ideas were developed over the decades with the aid of Underwood's five editions^(14)^ and Owen's four volumes on copper^(15)^. These works are still useful. According to Underwood, copper deficiency affects adversely the cardiovascular, gastrointestinal, haematopoietic, integumentary, musculoskeletal, nervous and reproductive systems.

According to Mills^(16)^, copper deficiency is the leading deficiency, worldwide, among nutritional diseases of agricultural animals. One wonders if a deficiency so common among domestic animals can be absent from their human associates. Emphasis here will be on diseases of the cardiovascular, musculoskeletal and nervous systems.

## Chemical and physiological evidence of nutritional deficiency

Data on organ/tissue analysis and metabolic pathways were collected from more than 60 medical publications revealing poor copper nutriture in more than 2500 people with diseases of the cardiovascular, musculoskeletal and nervous systems^(13)^. Data in Tables 2 and 3 reveal that nearly 6000 more people with common diseases have abnormally low copper concentrations or compromised metabolic pathways dependent on copper which are diagnostic of deficiency^(11)^. These impairments probably correspond to zones 3 or 4, potential or latent deficiency disease (Table 1). In addition to these tabulated diseases, two other copper deficiency syndromes are recognised.

**Table 2.** Low copper in organs, plasma, etc., in chronic diseases

	% of control value
Cardiovascular diseases	
Serum in CAD (81)^(17)^	93⋅0
Serum in CAD (30)^(18)^	83⋅6
Musculoskeletal diseases	
Serum in femoral neck fractures (46)^(19)^	78⋅0
Serum in age-related bone loss (225)^(20)^	72⋅5
Subnormal serum in elderly^a^ men (23⋅7 %) and women (32⋅9 %) (170)^(21)^	
Serum in postmenopausal O (576)^(22)^	84⋅4
Central incisor in low bone mineral density (50)^(23)^	53⋅1
Serum in female O (51)^(24)^	38⋅2
Nervous system diseases	
Frontal cortex in AD (8)^(25)^	56⋅5
Frontal cortex in dementia with Lewy bodies (6)^(25)^	52⋅2
Hippocampus in AD (15)^(26)^	51⋅1
Amygdala in AD (18)^(26)^	52⋅3
Seven brain regions in AD (9)^(27)^	52⋅8–70⋅2

Numbers in parentheses are the number of deficient people (total = 1285).

CAD, coronary artery disease; AD, Alzheimer's disease; O, osteoporosis.

a Age is related to heart disease risk.

**Table 3.** Low activities of copper-dependent enzymes in chronic diseases

	% of control value
Cardiovascular diseases	
Extracellular SOD in gestational diabetes^a^ (20)^(28)^	87⋅3
Plasma SOD in coronary artery ectasia (40)^(29)^	46⋅1
RBC SOD in coronary slow flow (20)^(30)^	65⋅3
RBC SOD in CAD (20)^(31)^	83
Extracellular SOD in Type 2 diabetes mellitus^a^ (31)^(32)^	68⋅0
Serum SOD in obesity^a^ (290)^(33)^	47⋅5
Plasma SOD in coronary angiography patients (766)^(34)^	75⋅3
Serum SOD in metabolic syndrome^a^ (3091)^(35)^	86⋅1
Musculoskeletal diseases	
Plasma SOD, RBC SOD in women with O (75)^(36)^	77⋅3, 66⋅6
Serum SOD in O (35)^(37)^	52⋅3
Nervous system diseases	
SOD in AD frontal cortex, cerebellum, and hippocampus (12)^(38)^	65 to 73
RBC SOD in AD (22)^(39)^	54⋅9 to 66⋅4
Caeruloplasmin specific activity in AD (84)^(40)^	94⋅7

SOD, superoxide dismutase; RBC, red blood cell; total patients = 4506.

a Conditions related to heart disease risk, see Table 2.

A new and severe neuropathy is being found increasingly in the last decade. It resembles that of pernicious anaemia, but it responds to copper rather than vitamin B_12_. Poor balance is the most common presenting complaint and probably is from cerebellar injury. The neuropathy seems rare enough to be published, but common enough that 10–15 cases can be reported from single clinics^(41,42)^. It may be as common as the neuropathy from vitamin B_12_ deficiency and may be the most important alternative in differential diagnosis of the latter^(43)^. If one excludes patients with obvious causes of copper deficiency such as bariatric surgery, dental adhesives high in zinc, haemochromatosis, iron or zinc supplementation, lead poisoning, malabsorption and soft drink excess, it seems that 20–40 % of the cases are of unknown origin and may be presumed to be dietary^(44)^.

Anaemia in copper deficiency has been studied for more than 90 years; the neuropathy can occur without it^(45)^; anaemia is a comparatively insensitive index of deficiency^(46)^. Copper deficiency can masquerade as myelodysplastic syndrome^(47–49)^.

Table 4 summarises beneficial effects of copper supplementation on metabolic pathways or functional systems of more than a thousand people. Some of the data are taken from an earlier collection^(13)^. These benefits conform to one of Golden's^(11)^ criteria of deficiency (above).

**Table 4.** Beneficial effects of copper supplementation

	% improvement
Copper alone	
Numerous premature heart beats vanished (3)^(50)^	100
Decrease in vertebral bone mineral density 0⋅51 units/yr. *v*. 3⋅75 for control (24)^(51)^	86⋅4
Increased RBC haemolysis time *in vitro* (26)^(52)^	6⋅1
Plasma homocysteine decreased (9)^(53)^	12⋅3
RBC SOD increased (16)^(54)^	11⋅2
Increased serum Cp (8)^(55)^	10⋅0
Increased serum diamine oxidase (8)^(55)^	78⋅8
Increased enzymatic Cp (35)^(56)^	23⋅4
Increased enzymatic RBC SOD (35)^(56)^	48⋅5
Vision improved from 20/400 to 20/25 (1)^(57)^	
Myelodysplastic syndrome was cured (2)^(58, 59)^	100
Copper with other nutrients	
Bone mineral density increased 1⋅48 % *v*. decreased 3⋅53 % for control (14)^(60)^	
LDL oxidation lag time *in vitro* increased (23)^(61)^	8
Reduction in vision decline (903)^(62, 63)^	28
Risk of death (903)^(62, 63)^	27
Left ventricular ejection fraction improved in heart failure (14)^(64)^	5⋅3

See Table 3; Cp, caeruloplasmin; LDL, low density lipoprotein; total patients = 1078.

Measurements of copper status on patients (Tables 2 and 3) ranged from 38 to 95 % of those made on people without the various diseases. All decreases were statistically significant. Copper supplementation also produced some cures (Table 4). Numerous similarities between animals deficient in copper and diseased people (below) also are evidence of human deficiency.

## Contemporaneous deficiency disease

### Ischaemic heart disease

The discovery^(65)^ that copper deficiency can increase plasma cholesterol in rats substantially was unprecedented. This observation has been confirmed in more than thirty independent laboratories, worldwide^(47)^. Thus, it fulfills two of Selye's^(66)^ criteria of important research by being both true and surprising.

This discovery began a search over the decades to determine the degree to which results were generalisable, Selye's third criterion. Gradually eighty anatomical, chemical and physiological similarities between animals deficient in copper and people with ischaemic heart disease were collected^(67)^ by reading Owen and Underwood (above) and by experimentation.

Four newly found similarities illustrate the method. Low activity of paraoxonase (also called homocysteine thiolactone hydrolase and PON1) associated with heart disease is described in several articles by Durrington and the Macknesses^(68)^; its activity is decreased in rats deficient in copper^(68)^.

This lactone is an irreversible inhibitor of lysyl oxidase which depends on copper to initiate cross-linking of collagen and elastin in arteries^(69)^. Borowczyk *et al.*^(70)^ found that urinary thiolactone predicts myocardial infarction. High thiolactone may impair arterial repair^(71)^.

Lavi *et al.*^(72)^ reviewed the presence of elevated isoprostanes in various disease states related to atherosclerosis. F_2_-isoprostanes are increased in rats deficient in copper^(73)^.

Tivesten *et al.*^(74)^ measured dehydroepiandrosterone (DHEA) in serum of nearly 2500 Swedish men and found that low levels predicted increased risk of coronary heart disease death after 5 years. Serum DHEA is decreased in rats deficient in copper^(75)^.

Glucose intolerance has long been associated with heart disease risk^(76)^. Glycated haemoglobin is associated with coronary artery stenosis and heart disease severity^(77,78)^. Rats deficient in copper have increased glycosylated haemoglobin^(79)^.

The copper deficiency theory is the simplest and most general explanation of the aetiology and pathophysiology of ischaemic heart disease. A considerable number of adults consume less copper than recommended amounts, probably because copper in the Western diet has decreased since the 1930s. There are interrelationships between copper deficiency and iron overload, fetal programming and homocysteine^(80)^. According to Kuhn, theories incorporating other theories contribute to scientific progress^(81)^.

### Osteoporosis

There can be no medical doubt that copper deficiency can cause osteoporosis^(47)^. Most of the twenty-nine relevant, medical publications describe deficient children^(46,82)^.

Strain^(83)^ hypothesised that diets low in copper may contribute to postmenopausal osteoporosis and noted that milk and milk products are among the poorest dietary sources of copper in confirmation of an earlier observation^(46)^. He suggested that some current recommendations for preventing osteoporosis may be detrimental. Graham and Cordano^(46)^ noticed that bone demineralisation precedes anaemia in copper deficiency.

Copper supplementation has been beneficial (Table 4). Several similarities between animals deficient in copper and people with osteoporosis have been tabulated^(82)^.

### Alzheimer's disease

It is hypothesised that copper deficiency is a plausible cause of Alzheimer's disease^(84)^. Patients are thinner than normal; weight loss precedes dementia and is associated with greater dementia and neurobehavioural symptoms. Nutritional compromise contributes to morbidity. Cytochrome oxidase depends on copper for activity; at least fourteen publications reveal decreased activity in brain of Alzheimer's patients. Brain copper and caeruloplasmin also are decreased. This hypothesis is the only one that explains why Alzheimer's disease occurs earlier and is more common in Down's syndrome.

Superoxide dismutase (SOD1) depends on copper for activity; its gene is on chromosome 21. This enzyme is elevated in Down's syndrome (trisomy 21) and is decreased in people with monosomy. It seems likely that people with Down's syndrome have a higher than average requirement for dietary copper because copper is incorporated into superoxide dismutase and is unavailable for other uses. Thus, Alzheimer's disease fulfills the first two of Golden's criteria (above) for deficiency.

## Research opportunities

Witte *et al.*^(64)^ supplemented thirty septuagenarian patients with chronic heart failure with either placebo or multiple micronutrients, including copper. Left ventricular ejection fraction and quality of life improved only in the supplemented group. The authors suggested that their regimen may be a first step towards identifying elements that can be eliminated without loss of benefit.

Confirmation and extension of these results with a follow-up trial can be relatively inexpensive and need not involve hundreds or thousands of participants often observed in heart disease research^(85)^. Some micronutrient doses were high (vitamins B_1_, B_6_, C and E, plus folate). A few groups of twenty patients can reveal if higher copper and lower vitamins affect results.

Decreasing thiamine to ten times the usual nutritional dose should protect against beriberi heart disease induced by some diuretics^(86)^. Vitamin C (500 mg/d) was more than three times the amount with a detectable diminution in copper metabolism^(87)^. Copper (1⋅2 mg/d) should be increased three or four-fold because deficient people should be supplemented with several times the usual nutritional dose^(47)^.

Studies of improved bone mineral density from an increased copper intake^(51,60)^ also should be repeated and extended. Calcium, and possibly zinc, may become limiting nutrients when copper alleviates the osteoporosis of middle age; other nutrients should not be ignored^(47)^. Witte *et al.*^(64)^ suggest that more than one nutrient may be important.

Several articles mention ocular lesions in copper deficient people very briefly^(88)^. Eyes and metabolism of deficient people should be examined carefully to characterise the lesions before and after supplementation.

Hundreds of articles associate the locus coeruleus with Alzheimer's disease. This locus has the highest concentration of copper in brain, and probably the body; it may have a place in memory^(89)^. If chemical analysis reveals lower copper in the locus of Alzheimer's disease patients than in similar people without dementia, a new approach to the disease will have been identified. Although autopsy specimens from people with Alzheimer's disease have been found low in copper, data on the locus were not found^(84)^.

Locus coeruleus volume decreases as Alzheimer dementia increases^(90)^. Tyrosine hydroxylase histochemistry in the locus can be used to assess early neuronal degeneration^(91)^. This enzyme falls by two-thirds in copper-deficient rat brain^(15)^. Measurement of this enzyme activity in Alzheimer's disease may be fruitful, particularly if accompanied by local copper measurements.

Research on these topics should include sensitive tests of copper deficiency such as enzymatic ceruloplasmin^(87,92)^.

## Conclusion

One can conclude from numerous medical articles that copper deficiency contributes to, and probably causes, Alzheimer's disease, ischaemic heart disease, some myelodysplastic syndrome and postmenopausal osteoporosis. These chronic diseases have low organ copper and impaired metabolic pathways dependent on copper. They improve with supplements containing copper. Thus, they exhibit classical characteristics of deficiency.

Published studies of improved bone mineral density in osteoporosis, improved left ventricular ejection fraction in heart failure, improved vision in macular degeneration and cure of myelodysplastic syndrome from an increased copper intake should be repeated and extended. Patients with Alzheimer's disease should be supplemented with copper to determine if memory decline can be delayed or reversed. Nutritional deficiency can be chronic. It is undesirable to make things complicated when a simple explanation will do^(84,93)^.
